# Nerve growth factor improves functional recovery by inhibiting endoplasmic reticulum stress-induced neuronal apoptosis in rats with spinal cord injury

**DOI:** 10.1186/1479-5876-12-130

**Published:** 2014-05-15

**Authors:** Hongyu Zhang, Fenzan Wu, Xiaoxia Kong, Jie Yang, Huijun Chen, Liancheng Deng, Yi Cheng, Libing Ye, Sipin Zhu, Xie Zhang, Zhouguang Wang, Hongxue Shi, Xiaobing Fu, Xiaokun Li, Huazi Xu, Li Lin, Jian Xiao

**Affiliations:** 1 Key Laboratory of Biotechnology and Pharmaceutical Engineering, Molecular Pharmacology Research Center, School of Pharmacy, Wenzhou Medical University, Wenzhou, Zhejiang 325035, China; 2 Department of Pharmacy, Cixi People’s Hospital, Cixi, Zhejiang 315300, China; 3 Institute of Hypoxia Research, School of Basic Medical Sciences, Wenzhou Medical University, Wenzhou, Zhejiang 325035, China; 4 Department of Orthopaedics, The Second Affiliated Hospital, Wenzhou Medical University, Wenzhou, Zhejiang 325035, China; 5 Medicine Research Center, Ningbo Medical Treatment Center Lihuili Hospital, Ningbo, Zhejiang 330200, China; 6 Institute of Basic Medical Sciences, Chinese PLA General Hospital, Beijing 100853, China

**Keywords:** Nerve growth factor, Endoplasmic reticulum stress, Spinal cord injury, Apoptosis, Akt/GSK-3β, ERK1/2

## Abstract

**Background:**

Endoplasmic reticulum (ER) stress-induced apoptosis plays a major role in various diseases, including spinal cord injury (SCI). Nerve growth factor (NGF) show neuroprotective effect and improve the recovery of SCI, but the relations of ER stress-induced apoptosis and the NGF therapeutic effect in SCI still unclear.

**Methods:**

Young adult female Sprague-Dawley rats’s vertebral column was exposed and a laminectomy was done at T9 vertebrae and moderate contusion injuries were performed using a vascular clip. NGF stock solution was diluted with 0.9% NaCl and administered intravenously at a dose of 20 μg/kg/day after SCI and then once per day until they were executed. Subsequently, the rats were executed at 1d, 3 d, 7d and 14d. The locomotor activities of SCI model rats were tested by the 21-point Basso-Beattie-Bresnahan (BBB) locomotion scale, inclined plane test and footprint analysis. In addition, Western blot analysis was performed to identify the expression of ER-stress related proteins including CHOP, GRP78 and caspase-12 both *in vivo* and *in vitro.* The level of cell apoptosis was determined by TUNEL *in vivo* and Flow cytometry *in vitro*. Relative downstream signals Akt/GSK-3β and ERK1/2were also analyzed with or without inhibitors *in vitro*.

**Results:**

Our results demonstrated that ER stress-induced apoptosis was involved in the injury of SCI model rats. NGF administration improved the motor function recovery and increased the neurons survival in the spinal cord lesions of the model rats. NGF decreases neuron apoptosis which measured by TUNEL and inhibits the activation of caspase-3 cascade. The ER stress-induced apoptosis response proteins CHOP, GRP78 and caspase-12 are inhibited by NGF treatment. Meanwhile, NGF administration also increased expression of growth-associated protein 43 (GAP43). The administration of NGF activated downstream signals Akt/GSK-3β and ERK1/2 in ER stress cell model *in vitro*.

**Conclusion:**

The neuroprotective role of NGF in the recovery of SCI is related to the inhibition of ER stress-induced cell death via the activation of downstream signals, also suggested a new trend of NGF translational drug development in the central neural system injuries which involved in the regulation of chronic ER stress.

## Introduction

Spinal cord injury (SCI) represents a severe health problem worldwide usually associated with life-long disabilities. SCI often affects individuals in their productive age, having an enormous social and economic impact. Traumatic SCI evolves through two phases: normally, the primary injury and the secondary injury which includes disturbances in ionic homeostasis, local edema, ischemia, focal hemorrhage, free radicals stress and inflammatory response [1]. Substantial research efforts are being devoted to limit the evolution of secondary damage through the development of neuroprotective measures [2]. Several reports have suggested that apoptosis plays a pivotal role in this secondary damage in animal models and in human tissue by causing progressive degeneration of the spinal cord [3]. However, the exact mechanism of cell death has not been fully clarified.

Endoplasmic reticulum is the important intracellular organelle for the synthesis and folding of secreted and membrane-bound proteins and the first site of the secretory pathway [4]. Some of the events triggered by the secondary injury, such as disturbance of calcium homeostasis, accumulation of unfolded or misfolded proteins and hypoglycemia, can trigger ER stress [5,6]. Consequently, to restore the proper function of ER and trigger the unfolded protein response (UPR), contribute to this adverse situation [7]. During SCI, prolonged ER stress without the cellular protective mechanisms by UPR eventually results in neural apoptosis [8,9]. Several apoptosis mediators are implicated in ER stress-associated cell death, such as glucose-regulated protein 78 (GRP78), the transcription activation of the C/EBP homologous transcription factor (CHOP) and the activation of ER-associated caspase-12 [10]. Accumulation of unfolded proteins in the ER lumen, inhibition of protein synthesis depletion of Ca^2+^ from ER stores, activation of CHOP/GADD153 expression and caspase-12, all these events suggest the crucial role of ER in neuronal cell apoptosis signaling after SCI [8,11]. Nevertheless, the latest study indicated that the deletion of the pro-apoptotic CHOP did not result in improvement of locomotor function after severe contusive spinal cord injury [12]. Although ER stress has been confirmed played important role in SCI, but the relative mechanism still need to further investigation.

Nerve growth factor (NGF) is a member of the neurotrophin family and an important regulator of neural survival, development, function and plasticity. In addition, NGF can also protect cells against oxidative stress or toxin-induced apoptosis [13,14]. The activation of the TrkA receptor and intracellular kinase pathways, including the phosphatidylinositol-3 kinase (PI3K)/Akt and mitogen-activated protein kinase (MAPK) pathways is involved in the neurogenesis and protective effect of NGF [15,16]. More recently, NGF has been demonstrated a role of promoting cell survival by counteracting apoptosis caused by ER stress [17,18]. It has been indicated that nerve growth factor (NGF) show neuroprotective effect and improve the recovery of SCI [19,20]. However, the molecular mechanism of NGF treatment in the recovery of SCI still undefined completely, especially the relations of ER stress-induced apoptosis and the NGF therapeutic effect in SCI has not been investigated clearly. The precise molecular mechanisms by which ER stress leads to cell survival/death remains enigmatic, with multiple potential participants described above, but little clarity about which specific death effectors dominate particularly in SCI, especially the mechanism and effective pathway in the treatment with exogenous neurotrophic factors.

In this study, we investigated the effect of exogenous NGF and the involvement of ER stress in a regional and time-dependent manner following clip compression-induce SCI. Our results indicated that the ER stress-induced apoptosis with the activation of CHOP, GRP78 and caspase-12 was involved in the early stage of SCI. NGF administration improved the locomotor function of SCI model rats, increased the neurons survival. The protective effect of NGF is related to the inhibition of ER stress-induced apoptosis, decreased the levels of CHOP, GRP78 and caspase-12, which also increased the expression of growth-associated protein 43 (GAP43). These results may certainly help in understanding the basic events involved in ER stress-mediated cell survival/death signaling pathways and the molecular mechanism in the recovery of SCI and other central nerve system diseases, which also contributes to the translational research of NGF in drug development.

## Materials and methods

### Cell culture and viability assay

PC12 cells were purchased from the Cell Storage Center of Wuhan University (Wuhan, China). PC12 Cells were cultured in Dulbecco’s Modified Eagle Medium (DMEM, Invitrogen, Carlsbad, CA) supplemented with heat-inactivated 10%fetal bovine serum (FBS, Invitrogen, Carlsbad, CA), 5% horse serum, and antibiotics (100 units/ml penicillin, 100 μg/ml streptomycin), incubated in a humidified atmosphere containing 5% CO_2_ at 37°C. PC12 cells were seeded on 96-well plates (5 × 10^3^ cells/well) and treated with different concentrations of ER stress activator, various doses of thapsigargin (TG, 0, 1μM, 2.5μM, 5μM, 10μM) for 24h. For determining the effect of NGF, 100ng/ml NGF was added 2 h prior to the addition of TG [21]. Cell viability was determined by MTT assays. 20 μl MTT (3-(4,5-dimethylthiazol-2-yl)-2, 5-diphe-nyltetrazolium bromide (5 mg/ml in PBS) was add to the cells for 4 h. Cells were washed with PBS (pH 7.4), and 150 μl DMSO was added to solubilize the formazan crystals. Fluorescence intensity was measured at 570 nm. Optimal conditions of 5μM TG and 100ng/ml NGF were used for the subsequent experiments.

To further evaluate the effect of PI3K/Akt and ERK1/2 activation on oxidative injury, cells were pretreated with specific inhibitors LY294002 (20 μM) and PD98059 (20 μM) 2 h before the addition of NGF as described previously [22]. Then analyze Cell signaling and cell survival. Pretreatment compounds were not removed from the media before successive treatment conditions. All experiments were performed in triplicate.

### Spinal cord injury and NGF administration

Young adult female Sprague-Dawley rats aged 8 weeks (220-250g) were purchased from Animal Center of Chinese Academy of Sciences, Shanghai, China. Animals were maintained for at least 7 d before the experiment in a temperature-regulated room (23°C-25°C) on a 12-h light/dark cycle and free to water and food. The protocol of the animal using and caring was conformed to Guide for the Care and Use of Laboratory Animals from National Institutes of Health and approved by the experimental procedures were approved by the Animal Care and Use Committee of Wenzhou Medical College. Animals were anaesthetized by an intraperitoneal injection of 10% chloralic hydras (3.5 ml/kg). Then rats were positioned on a cork platform. The skin was incised along the midline of back, and the vertebral column was exposed and a laminectomy was done at T9 vertebrae and moderate contusion injuries were performed using a vascular clip ( 2 min, 30 g forces, Oscar, China). Control group animals received the same surgical procedures, but impaction was not applied to the spinal cord. Postoperative care involved manual urinary bladder empty twice daily until they return of bladder function and administration of cefazolin sodium (50 mg/kg, i.p.).

NGF was purchased from Sigma (Sigma–Aldrich, St. Louis, MO, USA). The stock solution was diluted with 0.9% NaCl and administered intravenously at a dose of 20 μg/kg/day after 30 min of SCI and then once per day until they were executed. Control groups were received equivolumetric administration of NaCl at the corresponding times. Following treatment with either vehicle or NGF, animals were treated uniformly until the final analysis of the data. All experimental animals were received daily rehabilitation procedures, including passive mobilization of hind legs twice daily. Subsequently, the rats were executed at 1, 3, 7 and 14 d.

### Behavioral tests

In order to examine the locomotor function after injury, behavioral analyses were performed by trained investigators who were blind to the experimental conditions. Using the 21-point BBB locomotion scale, inclined plane test and footprint as described elsewhere [23] to evaluate open-field locomotion. BBB is a 22-point scale (scores 0-21) that systematically and logically follows recovery of hindlimb function from a score of 0, indicative of no observed hindlimb movements, to a score of 21, representative of a normal ambulating rodent.

The inclined plane test was performed via a testing apparatus [24]. The maximum angle at which a rat could retain its position for 5 sec without falling was recorded for each position, and averaged to obtain a single score for each animal. Footprint analysis was performed through dip the animal’s hindpaws with blue dye as described elsewhere [25]. The animal was allowed to walk across a narrow box (1 m long and 7 cm wide). The footprints were scanned, and digitized images were analyzed.

### Immunohistochemistry and histology

Sham and SCI rats (n = 6) were deeply re-anesthetized with 10% chloralic hydras (3.5 ml/kg, i.p.), and perfused with 0.9% NaCl, followed by 4% paraformaldehyde in 0.01M phosphate buffered saline (PBS, PH = 7.4) at 1, 3, 7 and 14 d. A T7–T9 spinal cord segment around the lesion epicenter was removed, post-fixed in cold 4% paraformaldehyde overnight, and embedded in paraffin. Transverse paraffin sections (5 μm thickness) were mounted in Poly-L-Lysine-coated slides for histopathological examination. The lesion epicenter stained with hematoxylin and eosin for HE staining. One stained with Cresyl Violet for Nissl stain following the instruction. Consecutive slides were immunostained. The slide were incubated in 3% H_2_O_2_ for 15 min and 80% carbinol for 30 min and then in blocking solution for 1 h at room temperature. Subsequently, the sections were incubated at 4°C overnight with the following primary antibodies: CHOP (1:150), GRP78 (1:200), and caspase-12 (1:2000, Santa Cruz Biotech, CA, USA). After triple washing in PBS, the sections were incubated with horseradish peroxidase-conjugated secondary antibodies for 2 h at 37°C. The reaction was stopped with 3, 3-diaminobenzidine (DAB). The results were imaged at a magnification of 400 using a Nikon ECLPSE 80i (Nikon, Japan). The optical densities and positive neuron numbers of CHOP, GRP78 and caspase-12 were counted at 5 randomly selected fields per sample in spinal anterior horn and quantification by Imagepro-Plus. Histology and immunohistochemistry for each marker were performed simultaneously in all spinal cord samples as well as negative controls without primary antibodies.

### Western blot analysis

For the *in vivo* protein analysis, a spinal cord segment (0.5 cm length) at the contusion epicenter was dissected at 1, 3, 7 and 14 d and soon stored at -80°C for western blotting. For protein extraction, the tissue was homogenized in modified RIPA buffer (50 mM Tris-HCl, 1% NP-40, 20 mM DTT, 150 mM NaCl, PH = 7.4) containing protease inhibitor cocktail (10 μl/ml, GE Healthcare Biosciences, PA, USA). The complex was then centrifuged at 12,000 rpm and the supernatant obtained for protein assay. For ER stress model *In vitro*, PC12 cells were lysed in RIPA buffer (25 mM Tris-HCl, 150 mM NaCl, 1% Nonidet P-40, 1% sodium deoxycholate, and 0.1% SDS) with protease and phosphatase inhibitors. The extracts above were quantified with bicinchoninic acid (BCA) reagents (Thermo, Rockford, IL, USA). We separated proteins (50 μg) on a 11.5% gel and transferred tem onto PVDF membrane (Bio-Rad, Hercules, CA, USA). The membrane was blocked with 5 % milk (Bio-Rad) in TBS with 0.05 % tween 20 for 1h and incubated with the antibodies: CHOP (1:300, Santa Cruz Biotechnology, CA, USA), GRP78 (1:300, Santa Cruz Biotechnology), caspase-12 (1:1000, Santa Cruz Biotechnology), caspase-3 (1:1000, Santa Cruz Biotechnology) in 5% milk in TBS with 0.05% tween 20 overnight. The membranes were washed with TBS for 3 times and treated with horseradish peroxidase-conjugated secondary antibodies for 1h at room temperature. Signals were visualized by ChemiDicTM XRS + Imaging System (Bio-Rad), and band uensities were quantified with Multi Gauge Software of Science Lab 2006 (FUJIFILM Corporation, Tokyo, Japan). We analyzed relative densities of the bands with Quantity One (version 4.5.2; Bio-Rad). Quantities of band densities were normalized using GAPDH.

### Apoptosis assay

DNA fragmentation *in vivo* was detected by one step TUNEL Apoptosis Asssy KIT (Roche, Mannheim, Germany). Transverse paraffin sections (5 μm thickness) were deparaffinized and rehydrated. Sections were treated with 10 μg/mL proteinase K at 37°C for 30 min, then incubated with 50 μL of TUNEL inspection fluid for 60 min before rinsed three times with PBS. Images were taken at × 400, using 488 nm wavelengths light for excitation and 530 nm for emission. Images were captured with a Nikon ECLIPSE Ti microscope (Nikon, Japan).

The apoptotic rates of the PC-12 cells treated with TG and NGF were measured using a PI/Annexin V-FITC kit (Invitrogen, Carlsbad, CA, USA), then analyzed by FACScan flow cytometer (Becton Dickinson, Franklin Lakes, NJ, USA) as the manual description.

### Immunofluorescence staining

The sections were incubated with 10% normal donkey serum for 1 h at room temperature in PBS containing 0.1% Triton X-100, followed by incubation with appropriate primary antibodies overnight at 4°C in the same buffer. The nuclears were stained with Hoechst 33258 (0.25 μg/ml) dye. For neurons and GAP43 detection, the following primary antibodies were used based on different targets: anti-NeuN (1:500, Millipore), anti-GAP43 (1:50, Santa Cruz, Biotechnology, Santa Cruz, CA). After primary antibody incubation, sections were washed for 4 × 10 min at room temperature, followed by incubation with Alexa Fluor594/647 donkey anti-mouse/rabbit, Alexa-Fluor488/594 donkey anti-rabbit/mouse, or Alexa-Fluor488/594 donkey anti-goat secondary antibody (1:500; Invitrogen Corporation, Carlsbad, CA, USA) for 1 h at room temperature. Sections were then washed with PBS containing 0.1% Triton X-100 for 4 × 10 min, followed by 3 × 5 min with PBS and briefly with water. All images were captured on Nikon ECLIPSE Ti microscope (Nikon, Tokyo, Japan).

### Statistical analysis

Data were expressed as mean ± SEM. Statistical significance was determined with Student’s t-test when there were two experimental groups. For more than two groups, statistical evaluation of the data was performed using One-way Analysis-of-variance (ANOVA) test, followed by Dunnett’s post hoc test with the values *P* < 0.05 considered significant.

## Results

### ER stress-induced apoptosis is involved in the early stage of SCI model *in vivo*

**Figure 1:**
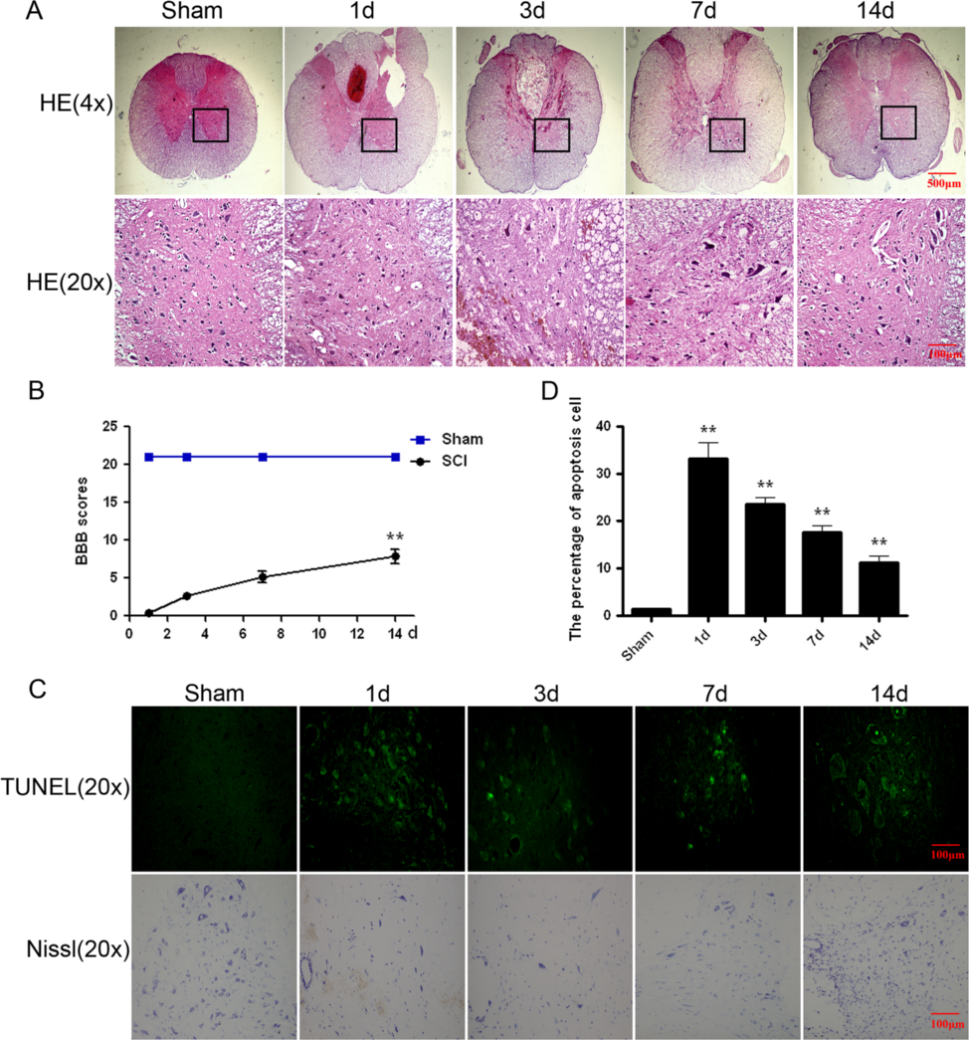
**Assessment of SCI model rats. A**. HE staining results of SCI rat at 1, 3, 7 and 14 d after contusion. **B**. The BBB scores of SCI model rat at 1, 3, 7 and 14 d after contusion. The score of the sham group was 21 points (meaning normal locomotion). ** P < 0.01 versus the sham group. Data are the mean values ± SEM, n = 6. **C**. TUNEL apoptosis assay of model rat spinal cord lesion. Immunofluorescence results of the TUNEL assay. Bright green dots were deemed positive apoptosis cell. Motor function was evaluated by Nissl staining. Big, blue dots were deemed as motor neuron. Magnification was 20 ×. **D**. The analysis of apoptosis cell 1, 3, 7 and 14 d after spinal cord injury lesions. The percentage of apoptosis was counted from 3 random 1 × 1 mm^2^ areas. ** P < 0.01 versus the sham group. Data are the mean values ± SEM, n = 6.

In order to evaluate the role of ER stress in SCI, we performed the SCI models as described above [26]. After SCI, animals showed dramatic and bilateral hind limb paralysis with no movement at all or only slight movements of a joint from the first hours post-injury when observed during open-field walking. Then treated with NGF at three dose (5, 10, 20 μg/kg) beginning 1 d after injury and then once daily for 14 consecutive days. Functional recovery was then evaluated for 2 weeks after injury using the BBB rating scale [23], inclined plane test [24], and footprint recordings [25]. We scored animal locomotor activity according to the BBB scale 1d after SCI, and the BBB scores showed that rats with SCI could no longer move (score 0 to 1) while the sham-operated rats walked normally (score 21). There were significant differences between both groups (*P* < 0.0001; Figure 1B). The locomotor function increased progressively during the experimental period. At 3 d and 7 d after contusion, the BBB scores were 3.67 ± 0.88 (*P* < 0.0001) and 8.00 ± 0.58 (*P* < 0.0001). Until 14 d after injury, the BBB scores were achieve 12 ± 0.88, which corresponds to slight step placement with weight support (Figure 1B).

The lesion center was characterized by the destruction of gray and white matter. The neurons in the spinal cord of the sham-operated group had normal morphology, with a clear cytoplasm, and uniform and clear nuclei. Compared with the sham operation group (1 d post-trauma), the gray matter of the SCI group exhibited large hemorrhages. The motor neurons in the anterior horn were shrunken or had pale homogenous cytoplasm compared with the sham group, consistent with ischemic change. Progressive destruction of the dorsal white matter and central gray matter tissue was found 3 d post-injury. The lesion segments displayed hemorrhagic necrosis, neuron loss, karyopyknosis, and infiltrated polymorphonuclear leukocytes and macrophages. In the segments collected 7 d post-injury and 14 d post-injury, there was minimal hemorrhaging and some neuron regeneration, but demyelination appeared as well as numerous cavities (Figure 1A). The cell apoptosis in the spinal lesions were detected by TUNEL staining and the bright green dots were deemed as TUNEL-positive cells in the lesions. Sham group showed no apoptosis positive cells. The numbers of TUNEL-positive cells increased significantly 1d after injury and were maximal at 3 d, then decreasing gradually (Figure 1C, D). The effect of SCI on the number of motor neurons in the spinal cord was investigated by Nissl staining. As shown in Figure 1C, SCI rat showed an extensive loss of large anterior horn cells at 1d and 3 d. In contrast, motor neurons were increased in the anterior horns at 7 d. At 14 d group compared with sham group, motor neurons were remarkably preserved in the anterior horns.

**Figure 2:**
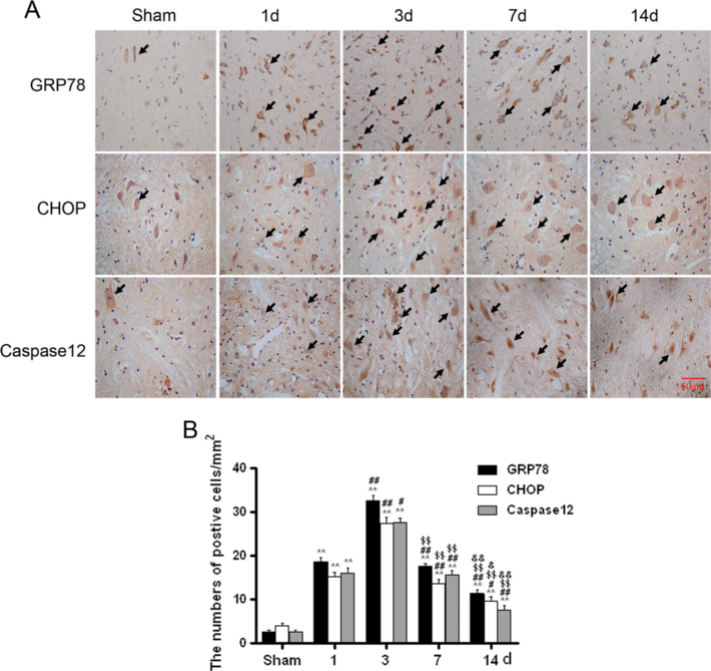
**ER stress-induced apoptosis was involved in the early stage of SCI. A**. Immunohistochemistry for GRP78, CHOP and caspase-12 in sham, 1, 3, 7 and 14 d after spinal cord injury lesions groups. **B**. Analysis of the positive cells of the immunohistochemistry results. * represents P < 0.05 versus the sham group, ** P < 0.01 versus the sham group, ^#^ P < 0.05 versus the 1d group, and ^$^ P < 0.05 versus the 3 d group, & P < 0.05 versus the 7 d group. Data are the mean values ± SEM, n = 6.

We next investigate the molecular mechanism of ER stress-induced apoptosis in the SCI, immunohisochemistry staining was applied firstly. It was found that GRP78, CHOP, caspase-12 positive cells were expressed in both gray matter and white matter, and the staining was more intense in the gray than white matter (Figure 2A). The numbers of CHOP, GRP78, caspase-12 positive cells and optical density increased significantly, and were all maximal at 3 d (Figure 2A, B). Thereafter, the number of positive cells and optical density gradually reduced but were still observed after 7d contusion (Figure 2A, B). Until the 14d after injury, CHOP, GRP78, caspase-12 positive cells next to normal.

### NGF increases the survival of neurons and improves the recovery of SCI

**Figure 3:**
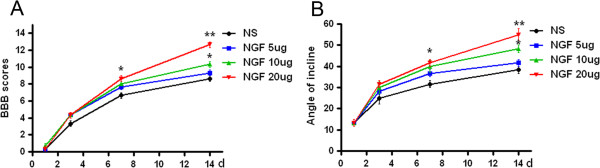
**BBB scores and inclined plane test of different concentration of NGF treated model rats. A**. BBB scores and **B**. inclined plane test after spinal cord injury. *P < 0.05 versus sham group, **P < 0.01 versus sham group.

**Figure 4:**
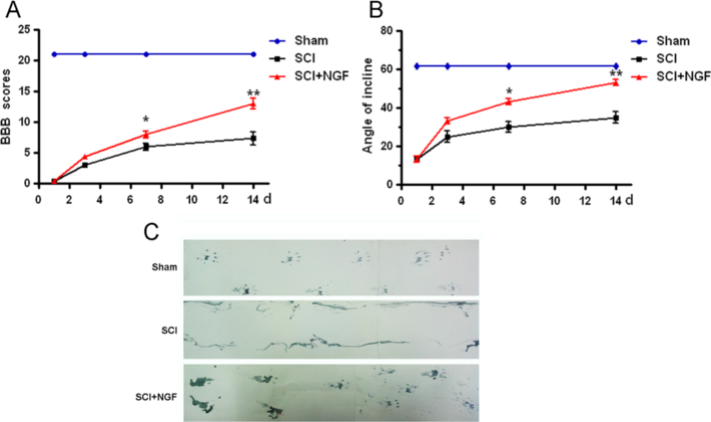
**NGF improves the locomotor activity of SCI rats. A**. The BBB scores and **B**. incline plane test of sham, SCI group and SCI rat treated with NGF group. * P < 0.05 versus the SCI group, and ** P < 0.01 versus the SCI group, n = 6. **C**. Representative footprints obtained from each group at 14 days after SCI show that NGF treated rats display fairly consistent weight-support plantar stepping and very little toe dragging. In contrast, vehicle control rats show consistent dorsal stepping and extensive toe dragging.

To evaluate the therapeutic effect of NGF on SCI, model rats were treated with NGF by tail intravenous injection. To determine the dosage of NGF firstly, three dosage of NGF (5, 10 and 20 μg/kg/d) was used for administration. As show in BBB scores (Figure 3A) and the inclined plane test (Figure 3B), the protective effect of NGF reached a maximum at 20 μg/kg/d. The BBB locomotor rating scores of SCI group were assessed at 1, 3, 7 and 14 d post-surgery. The hindlimbs were paralyzed immediately after injury, and the rats recovered extensive movement of hindlimbs within 7-14 days after injury. NGF treatment after injury significantly increased the hindlimb locomotor function, as assessed by BBB scores (Figure 4A). We previously discovered that nerve growth factor (NGF) reduced the SCI. As shown in Figure 4, NGF improve the BBB locomotor function compared to the SCI group at 7 d (P = 0.0132), and the effects become obviously at 14 d after contusion (P = 0.0011). At 14 d after injury, the 20 μg/ml NGF group reached at 11.67 ± 0.33, while the control group at 8.667 ± 0.33.

The angle of incline, determined 2 weeks after injury, was also significantly higher in NGF treated rats compared with the vehicle control group (*P* < 0.01, Figure 4B). As shown in Figure 4C, footprint analyses for NGF treated rats at 14 d after SCI disclosed fairly consistent hindlimb coordination and very little toe dragging. These findings were comparable to those in the sham control animals. By contrast, the footprints obtained from vehicle-treated animals showed inconsistent coordination and extensive drags as revealed by ink streaks extending from both hindlimbs (Figure 4C).

**Figure 5:**
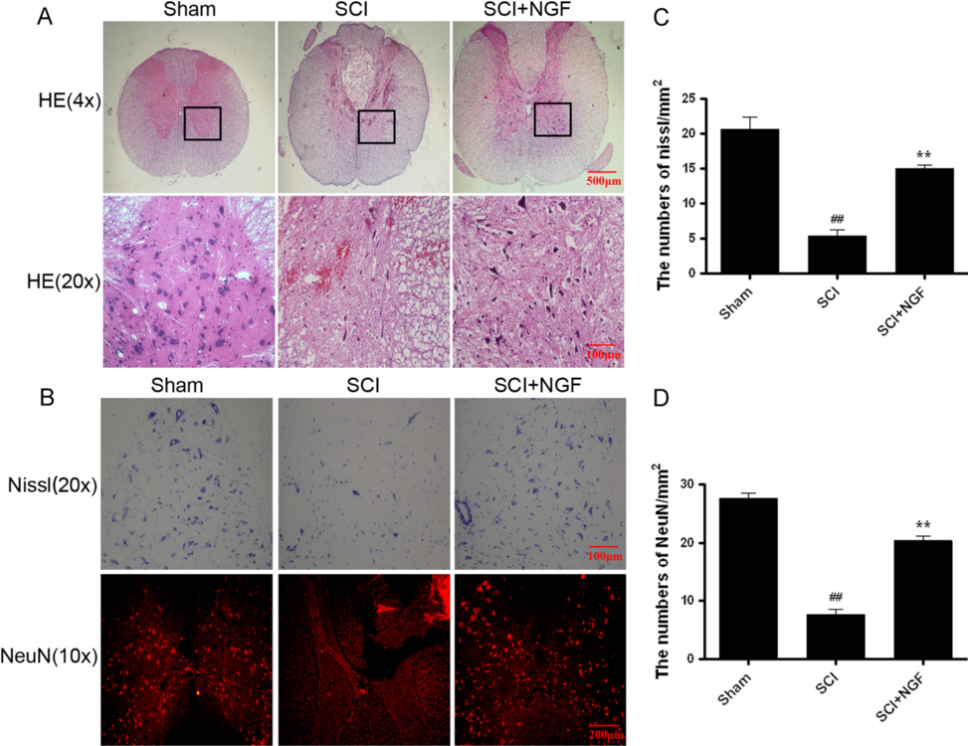
**Treatment with NGF improves the recovery of SCI and the survival of neurons. A**. HE staining results of the sham, SCI group and SCI rat treated with NGF group. **B**. NeuN staining and Nissl staining results of the sham, SCI group and SCI rat treated with NGF group. **C**. The analysis of Nissl body after spinal cord injury lesions in different groups. ^##^ represents P < 0.01 versus the sham group, ** P < 0.01 versus the SCI group. n = 6. **D**. Analysis of the positive neurons of the NeuN staining results. ^##^ represents P < 0.01 versus the sham group, ** P < 0.01 versus the SCI group. n = 6.

HE staining results of the spinal cord samples from different groups were shown in Figure 5A. Compared with sham operation group, progressive destroy of the dorsal white matter and central gray matter tissue were found in the 7 d SCI group. Compared with the SCI group, NGF treatment showed significant protective effect with less necrosis, karyopyknosis, infiltrated polymorphonuclear leukocytes and macrophages. The effect of NGF on the number of motor neurons in the spinal cord was also investigated. As shown in Figure 5B, the vehicle control group showed an extensive loss of large anterior horn cells. In contrast, motor neurons were remarkably preserved in the anterior horns in rats treated with NGF (20 μg/kg/d) compared with vehicle control rats. No significant difference was observed in rats between sham group and NGF group.

To further confirm the protective effect of NGF, we investigated the survival of neurons directly by immunofluorecence staining. As shown in Figure 5B, spinal cord neurons in both of the white and gray matter tissue were marked by neuronal marker NeuN. The positive staining cells decreased significantly after SCI at 7 d, and increased by NGF treatment (*P* < 0.0001). All of these data indicated that NGF administration shown protective effect on neuronal cells and improved the recovery of SCI significantly.

### NGF inhibits ER stress induced apoptosis and up-regulates the neuroprotective factors

**Figure 6:**
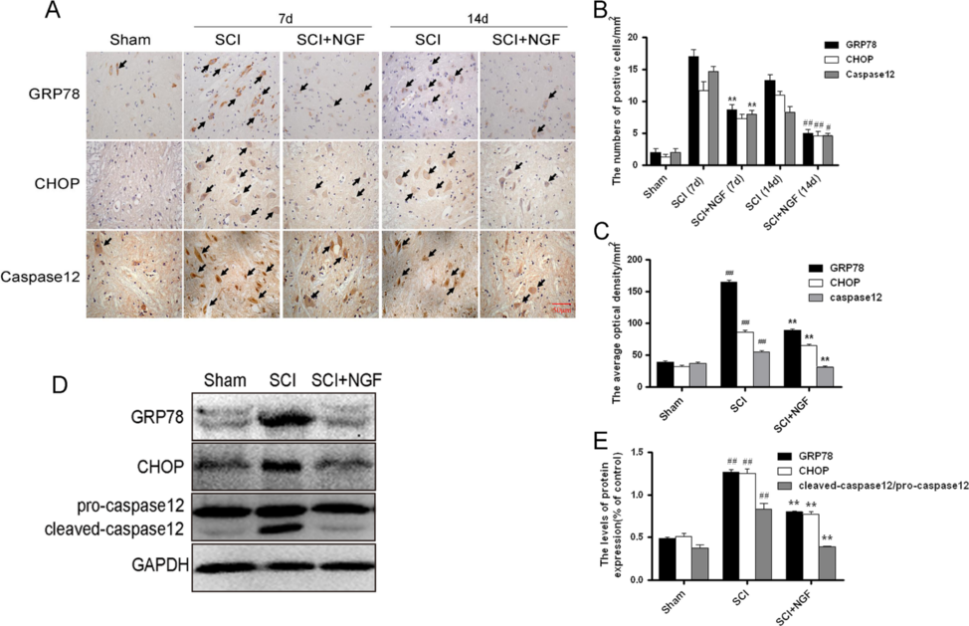
**NGF administration inhibits the expressions of ER stress-induced apoptosis response proteins, GRP78, CHOP and caspase-12. A**. Immunohistochemistry for GRP78, CHOP and caspase-12 in the sham, 7d and 14d after spinal cord injury lesion and NGF treatment 7 d and 14d after injury groups. **B**. Analysis of the positive cells and **C**. optical density of the immunohistochemistry results. ** P < 0.01 versus the SCI (7d) group, ^#^ represents P < 0.05 versus the SCI(14d) group, Data are the mean values ± SEM, n = 6. **D**. Protein expressions of GRP78, CHOP and caspase-12 for the sham, SCI and NGF treatment groups. GAPDH was used as the loading control and for band density normalization. **E**. The optical density analysis of GRP78, CHOP, and caspase-12 protein. ** P < 0.01 versus the sham group. Data are the mean values ± SEM, n = 6.

To illustrate whether the molecular mechanism of NGF is related to the regulation of ER stress, the protein expression of ER stress-induced apoptosis were detected by immunohistochemistry staining and Western blot. As shown in Figure 6A and Figure 6B, ER stress-induced apoptosis proteins (CHOP, GRP78 and caspase-12) positive cells and optical density gradually reduced by NGF administration after 7d contusion. The protein expression was also detected by Western blot, it was found that the levels of CHOP, GRP78 and caspase-12 protein decreased by the treatment of NGF, compared with the SCI group after 7 d contusion (Figure 6D, E). Until 14 days, the positive cells and protein expression all tend to normal level.

**Figure 7:**
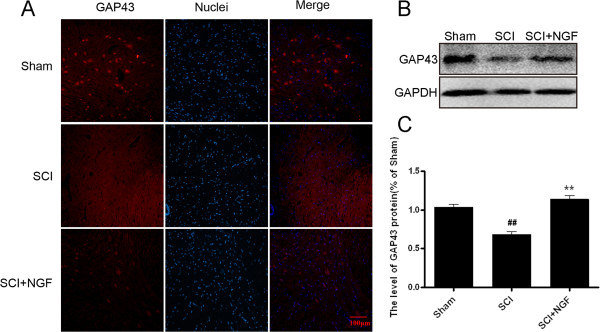
**NGF treatment increases the level of GAP43 in spinal cord lesions. A**. Immunofluorescence staining results of GAP43; the nuclear is labeled by Hoechst (blue), the neurons with obvious GAP43 signals are labeled by bright right dots, magnification was 20 ×. **B**. The protein expressions of GAP43 in sham, SCI rats and SCI rats treated with NGF groups. GAPDH was used as the loading control and for band density normalization. **C**. The optical density analysis of GAP43 protein. ** P < 0.01 versus the SCI group, and ^##^ represents P < 0.01 versus the sham group. Data are the mean values ± SEM, n = 6.

The protein GAP43 is expressed in developing and regenerating neurons which is often used to score the condition of neural regeneration. The levels of GAP43 were detected by immunofluorecence staining, it was found that the positive red fluorescence signal in the cytoplasm enhanced obviously in the NGF administration group compared with the SCI group at 7d contusion (Figure 7A). As shown in Figure 7B and C, Western blot analysis of GAP43 protein also demonstrated that the expression of GAP43 increased in the NGF treatment group, compared with SCI group after 7d contusion (*P* =0.0019), which was consistent with the results of immunofluorecence staining analysis.

**Figure 8:**
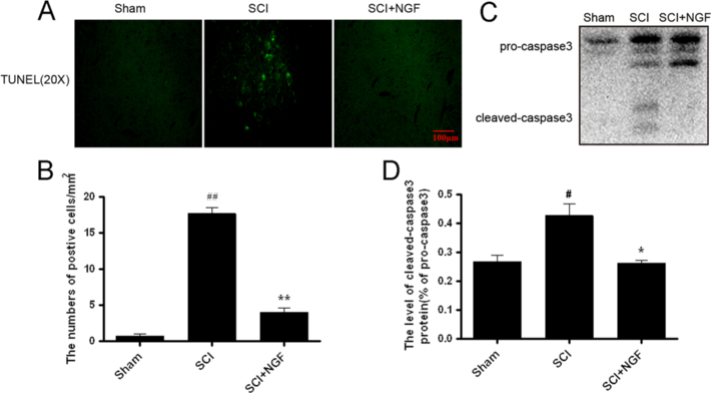
**NGF decreases the level of apoptosis in spinal cord lesions. A**. TUNEL apoptosis assay of model rat spinal cord lesions. Immunofluorescence result of the TUNEL assay. Bright green dots were deemed positive apoptosis cell, magnification was 20×. **B**. The analysis of apoptosis cell of the sham, SCI and NGF treatment groups. The percentage of apoptosis was counted from 3 random 1 × 1 mm^2^ areas. **C**. Protein expressions of caspase-3 for the sham, SCI and NGF treatment groups. **D**. The optical density analysis of caspase-3 protein. * P < 0.05 versus the SCI group, and ^#^ P < 0.05 versus the sham group. ** P < 0.01 versus the SCI group, and ^##^ P < 0.01 versus the sham group. Data are the mean values ± SEM, n = 6.

To investigate the effect of NGF on cell death after SCI, we performed TUNEL staining with sections obtained at 7 d after injury, the TUNEL-positive cells were obviously decreased in the NGF-treated rat compared with the vehicle-treated rat (Figure 8A). When the TUNEL-positive cells were counted, the number of TUNEL-positive cells was significantly lower in the NGF treated rats compared to the vehicle-treated rat (*P* =0.0019) (Figure 8B). In the Western blot analysis, the expression of Caspase3 protein was significantly increased in the NGF-treated rat and the vehicle-treated rat compared with in the sham controls (Figure 8C, D). In addition, the activated Caspase3 expression in the NGF-treated rat was relatively lower than that in the vehicle-treated rat.

### The protective role of NGF is related to the activation of downstream signal pathways PI3K/Akt/ GSK-3β and ERK1/2

**Figure 9:**
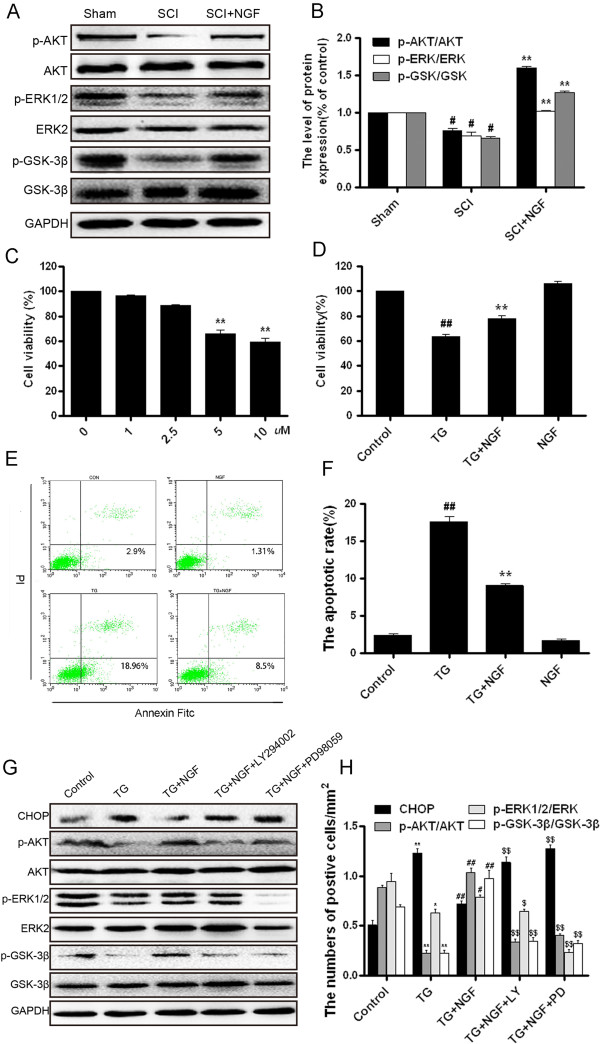
**PI3K/Akt/GSK-3β and ERK1/2 signals are involved in the protective effect of bFGF both in SCI rats and PC12 cells under stress. A**. The protein expressions of p-Akt/Akt, p-ERK/ERK, p-GSK-3β/GSK-3β in the sham, SCI model and SCI rat treated with bFGF groups. **B**. The optical density analysis of p-Akt/Akt, p-ERK/ERK, p-GSK-3β/GSK-3β protein. ** P < 0.01 versus the sham group, and ^#^ P < 0.05 versus the SCI group. Data are the mean values ± SEM, n = 6. **C**. MTT results of the different concentrations of TG-treated PC12 cells. **D**. MTT result of NGF-treated PC12 cells induced by TG. **E**. FACScan result of PI/Annexin V-FITC staining for cell apoptosis analysis. **F**. Statistical result of apoptosis rate in PC12 cells treated with TG and NGF. ** P < 0.01 versus the control group, and ^#^ P < 0.05 versus the TG group. Data are the mean values ± SEM, n = 3. **G**. The protein expressions of CHOP, GRP78, p-Akt, p-ERK1/2, p-GSK-3β in ER stress-induced apoptosis PC12 cells treated with NGF and different inhibitors. GAPDH was used as the loading control and for band density normalization. **H**. The optical density analysis of CHOP, GRP78, p-Akt, p-ERK1/2 and p-GSK-3β protein. * P < 0.05 versus the control group, ^#^ represents P < 0.05 versus the TG group, ^$^ P < 0.05 versus the TG + NGF group. Data are the mean values ± SEM, n = 6.

It has been indicated that PI3K/Akt/GSK-3β and ERK1/2 pathways are the main downstream signals which activated by NGF, two pathways are related to the cell survival, differentiation and migration [27,28]. We next detected whether the protective effect of NGF in the recovery of SCI also involved in the activation of these two signal pathways. Western blot analysis demonstrated that the expressions of the phosphoryations of Akt (p-Akt), ERK1/2 (p-ERK1/2) and GSK-3β (p-GSK-3β, downstream of Akt signal) decreased after SCI contusion. The decreases of phosphoryations were recovered by NGF treatment at 7 d (Figure 9A, B). These data indicated that both of the PI3K/Akt and ERK1/2 signals were involved in the role of NGF in the recovery of SCI.

### The activation of downstream signals is crucial for the protective effect of NGF in the chronic ER stress model *in vitro*

To further confirm the role of NGF in the ER stress-induced apoptosis *in vitro*, we used ER stress activator TG treated PC-12 cells to replicate the apoptosis model. As shown in Figure 9C and D, cell viability decreased with the increase of TG concentration (*P* = 0.0019), combination with NGF partially increased cell viability compared with TG group (*P* = 0.0004). Cell apoptosis was analyzed by FACScan with PI/Annexin V-FITC staining, it was found that NGF inhibited the apoptosis induced by TG in PC-12 cells, compared with TG group (*P* = 0.0003, Figure 9E, F).

To investigate the molecular mechanism of NGF in the ER stress-induced cell apoptosis model *in vitro*, two signal inhibitors LY294002 for PI3K/Akt and PD98059 for ERK1/2 were added. Both of these inhibitors have no effect on cell death when used respectively [29,30]. The activations of CHOP, p-Akt, p-ERK1/2 and p-GSK-3β were determined by Western blot analysis. As shown in Figure 9G and H, the activation of CHOP by TG treatment was inhibited by NGF addition, but the protective effect of NGF was abolished by LY294002 and PD98059, the expression of CHOP increased significantly by the inhibitors combination, compared with NGF treatment group (*P* = 0.0049 and *P* = 0.0046). Meanwhile, the levels of p-Akt, p-ERK1/2 and p-GSK-3β increased by NGF treatment and decreased by inhibitors addition. All of these data demonstrated the protective role of NGF in the ER stress-induced apoptosis is related to the activation of downstream signals PI3K/Akt and ERK1/2 both *in vitro* and *in vivo.*

## Discussion

The failure of axons to regenerate following CNS trauma results from decreased intrinsic properties of the neurons [31], the most predominant features which related to the recovery of SCI are the absence of neurotrophic factors and the presence of inhibitory factors in the environment [32,33]. In this study, our goal was to identify the cellular and molecular changes after acute spinal cord injury and NGF treatment that improve locomotor function of model rats.

Previous studies have demonstrated a variety of crucial functions of NGF in the nervous system, which is associated with central neuronal plasticity. As a member of neurotrophic factors, NGF was discovered as a molecule that stimulates the survival and maturation of developing neurons in the peripheral nervous system and has later been shown to protect adult neurons in the degenerating mammalian brain [34,35]. Zhang et al demonstrated that treatment with recombinant DNA vaccine promotes functional recovery in spinal cord hemisected adult rats, associated with expression of endogeous NGF significantly upregulated [36]. Besides, latest study investigated the possible protective actions of melatonin on SCI-induced damage and urinary bladder dysfunction, reduce of NGF levels due to SCI were restored by melatonin treatment, suggest that melatonin reduces SCI-induced tissue injury and improves bladder functions through its effects on oxidative stress and NGF. Administration with recombinant adenovirus encoding NGF along saphe nous branch in transected femoral nerve, NGF acted as a guidance molecule to promote branch point entrance of sensory axons into the saphenous branch, significantly increased the accuracy of saphenous branch neurons reinnervation [37]. In our study, treatment with exogenous NGF increased the locomotor function progressively during the experimental period. Increased survival of neurons markedly, increased the level of GAP43 which is considered a crucial component of an effective regenerative response in the nervous system, indicated that exogenous NGF administration shown protective effect and improved the recovery of SCI.

It is well known that SCI is a process involving various self-destructive processes that occur by a variety of factors based on disturbances in ionic homeostasis, local edema, focal hemorrhage, excitotoxicity, presence of free radicals and free fatty acids [38,39]. Certain studies have indicated that neuronal and glial cell apoptosis plays a role in SCI and that the inhibition of neuronal and oligodendroglial apoptosis may be a therapeutic strategy [40,41]. The results from the present study demonstrated that the pathological changes of the neurons and TUNEL-positive cells increased in the injured spinal cord, which led to neuronal and glial cell loss, and finally induced the impairment of locomotor activity according to the BBB scale. Certain studies, have suggested that the ER stress-signal may have a direct role in promoting cell death in neuronal injury diseases [42,43]. CHOP plays a critical role in ER stress-induced apoptosis, and it is believed to play a central role in ER stress-induced cell death, has been implicated in mediating neurodegeneration in animals with Alzheimer's disease [44]. CHOP activation has been observed in neurons undergoing apoptosis due to perturbations in ER calcium levels in neurotoxin model of parkinsonism *in vivo*[45]. Following spinal cord injury, rats deficient in CHOP signaling show increased spared white matter and enhanced locomotor recovery by 6 weeks. At 24 hours after SCI, ATF4 and CHOP are upregulated in under perfused microvessels. The expression increased levels of CHOP in the rats with SCI revealed that CHOP-mediated ER stress-induced apoptosis may be involved in SCI [46]. *In vitro*, it has also been indicated that NGF could protect PC12 cells against ER stress induced by TG, the role of pre-treatment NGF is related to the inhibition of Bax translocation from cytosol to the mitochondria, which led to loss of mitochondrial transmembrane potential, cytochrome c release, activation of caspases and apoptosis [21]. In this study, we demonstrated that ER stress-induced apoptosis was involved in the responses of SCI, the levels of related proteins including CHOP, GRP78 and caspase-12 protein increased obviously and decreased by treatment of NGF after 7d contusion *in vivo*, which also been detected in PC12 cell injury models induced by TG, suggested the neuroprotective effect of NGF was related to the inhibition of chronic ER stress-induced apoptosis.

Early study has demonstrated that the role of NGF is related to tyrosine kinase A (TrkA), and activation of downstream signals [47], including PI3K/Akt/GSK-3βand MEK/ERK1/2 [48-50]. Study has observed a time-dependent and dose-dependent effect of NGF on the PI3K/Akt and MAPK/ERK pathways, which also been involved in the other neurothrophic factors such as basic fibroblast growth factor in our previous studies of the recovery of SCI [26]. It has reported that localized microdomains of axonal PI3K activity drive the activity of axonal F-actin patches which essential for the formation of filopodia and branches from the axon, and show that NGF increases the formation of axonal filopodia by signaling through PI3K-Akt [51]. Other latter findings are consistent with the key role of PI3K and ERK signals in sensitization of TRPV1 by NGF and may explain the previously published observation that adult, but not neonatal, rat dorsal root ganglion neurons are sensitized by NGF [52]. Besides, NGF protects dorsal root ganglion neurons from oxaliplatin by modulating JNK/Sapk and ERK1/2, the findings assess the validity of MAPKs as the target of neuroprotective therapies during chemotherapeutic treatment. Moreover they also describe a double role for ERK1/2, depending on cellular stimulation, since it mediates neuronal apoptosis after oxaliplatin exposure [53]. When a specific inhibitor of PI3K, LY294002, was added to glucose analog 2-deoxy-D-glucose plus NGF-treated PC12 cells, both the effects of NGF on 2DG-induced apoptosis and GRP78 expression were significantly diminished [19]. In our study, the protective effect of NGF was also abolished by specific inhibitors LY294002 and PD98059, confirmed that the protective role of NGF here is related to the activation of downstream signals PI3K/Akt/GSK-3β and ERK1/2 in ER stress-induced apoptosis. In the following study, to further confirmed the role of NGF and the relations with chronic ER stress in the recovery of SCI model, siRNA of CHOP or ATF4 should be applied, which may contributes to the evidences of NGF protection through regulation of ER stress in neuronal cell death *in vivo*. Even so, over the past 30 years neurotrophic factors have generated considerable excitement for their potential as therapy for a wide variety of neurological disorders, for which there is currently no treatment. NGF appeared to be efficacious in two phase II clinical trials, but failed in a large scale phase III trial, to explore the appropriate therapeutic time window and special drug delivery system combined with factors still need long-term study. It also need to be addressed that NGF is not stable enough which is easy to be degradated by various enzymes *in vitro*, resulting in the loss of biological activity. So the combination with other drugs or delivery systems to increase its stability and smoothly through the spinal cord barrier may contributes to the functions of NGF. While the therapeutic potential of NGF has been well-recognized for decades, attempts to translate that potential to the clinic have been disappointing, largely due to significant delivery obstacles. Moreover, the mechanism of traumatic CNS injury diseases also undefined clearly, this study lays the ground work for future translational confidence of NGF in CNS diseases, especially the relations to ER stress. Collectively, the translational application study of NGF may not only focus on the effect in the neurogenesis, the combination with special biomaterials which can improves the efficiency and prolongs the effective time should also be addressed.

In conclusion, our research demonstrated that treatment with exogenous NGF increased the survival of neurons in the spinal cord lesions and improved the functional recovery of acute spinal cord injury model rats. Inhibition of ER stress induced apoptosis and increase of neuroprotective factor GAP43 is involved in the role of NGF in the neuronal cell death both *in vivo* and *in vitro*. These data demonstrate that therapeutic strategies targeting on chronic ER stress with NGF which also plays neurotrophic effects may be suitable for the therapy of central nervous system injuries, the effective combination with special drug delivery system or other factors still need to further investigations.

## Competing interests

The authors declare no conflict of interest.

## Authors’ contributions

JX and LL conceived and designed the experiments. HYZ, FZW, XXK, JY, HJC, LCD, YC, LBY and SPZ performed the experiments. ZGW, HXS JX analyzed the data. XZ, XBF, HZX and XKL contributed reagents/materials/analysis tools. HYZ, JX wrote the paper.
