# Correction to: Nerve growth factor improves functional recovery by inhibiting endoplasmic reticulum stress-induced neuronal apoptosis in rats with spinal cord injury

**DOI:** 10.1186/s12967-021-02901-7

**Published:** 2021-10-25

**Authors:** Hongyu Zhang, Fenzan Wu, Xiaoxia Kong, Jie Yang, Huijun Chen, Liancheng Deng, Yi Cheng, Libing Ye, Sipin Zhu, Xie Zhang, Zhouguang Wang, Hongxue Shi, Xiaobing Fu, Xiaokun Li, Huazi Xu, Li Lin, Jian Xiao

**Affiliations:** 1 Key Laboratory of Biotechnology and Pharmaceutical Engineering, Molecular Pharmacology Research Center, School of Pharmacy, Wenzhou Medical University, Wenzhou, 325035 Zhejiang China; 2 Department of Pharmacy, Cixi People’s Hospital, Cixi, 315300 Zhejiang China; 3 Institute of Hypoxia Research, School of Basic Medical Sciences, Wenzhou Medical University, Wenzhou, 325035 Zhejiang China; 4 Department of Orthopaedics, The Second Affiliated Hospital, Wenzhou Medical University, Wenzhou, 325035 Zhejiang China; 5 Medicine Research Center, Ningbo Medical Treatment Center Lihuili Hospital, Ningbo, 330200 Zhejiang China; 6 Institute of Basic Medical Sciences, Chinese PLA General Hospital, Beijing, 100853 China

## Correction to: J Transl Med 12:130 (2014) https://doi.org/10.1186/1479-5876-12-130

The original publication of this article [1] contained 4 errors in Figs. 1a, 5a, 7b, c. The wrong figures were used for the experimental animals and experimental groups. The correct figures are published in this correction article along with an updated caption. The full figures and captions can be accessed via the original article.

**Fig. 1:**
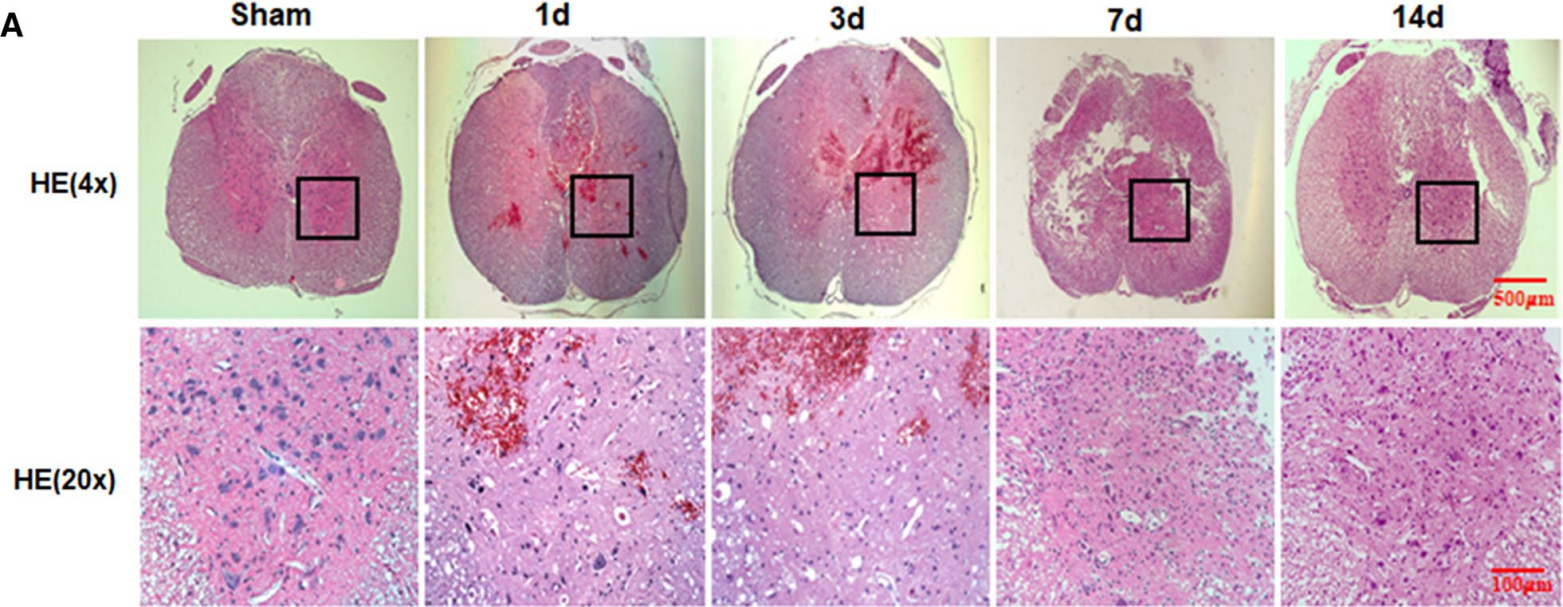
**A**. HE staining results of SCI rat at 1, 3, 7 and 14 d after contusion

**Fig. 5:**
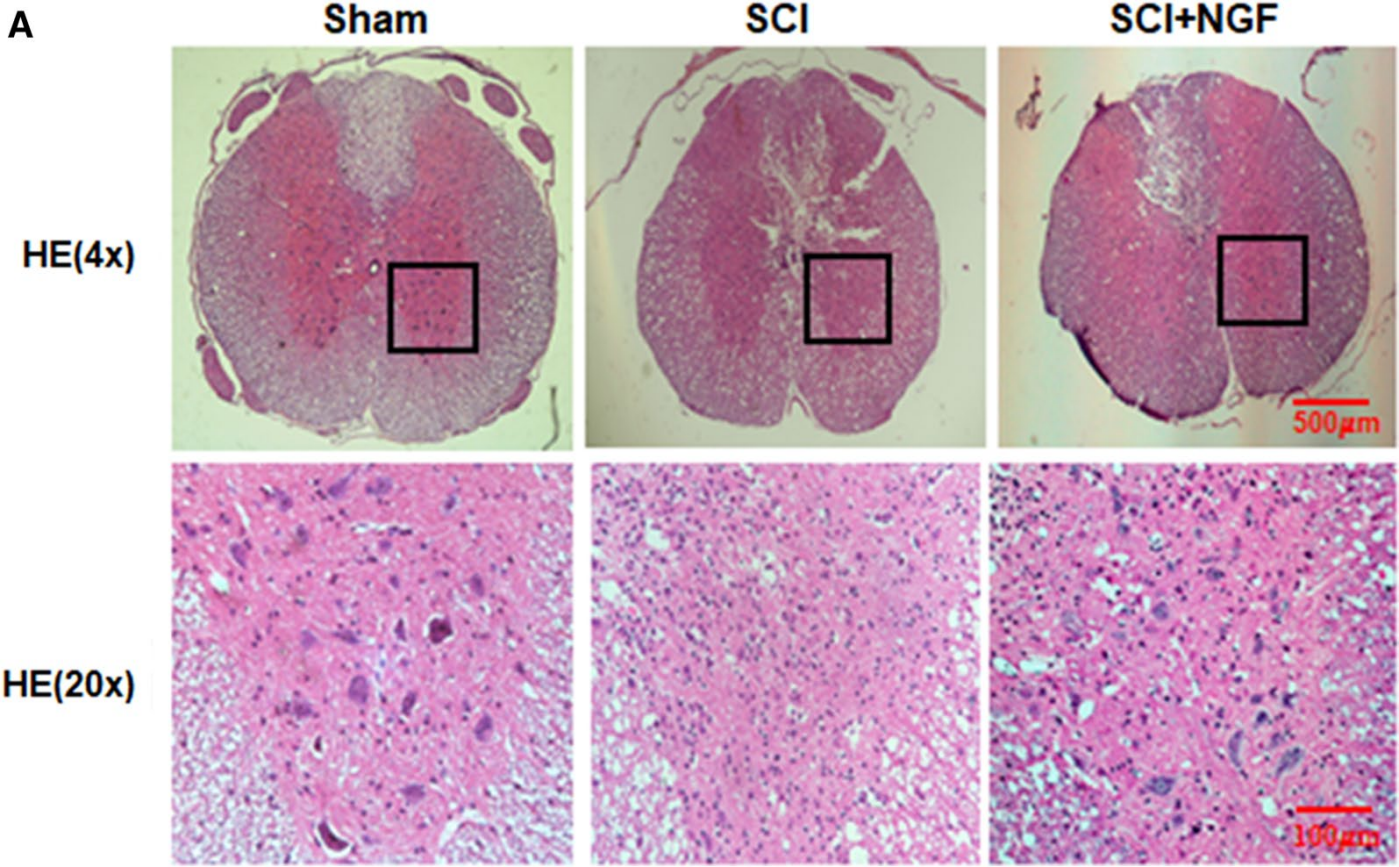
**A**. HE staining results of the sham, SCI group and SCI rat treated with NGF group

**Fig. 7:**
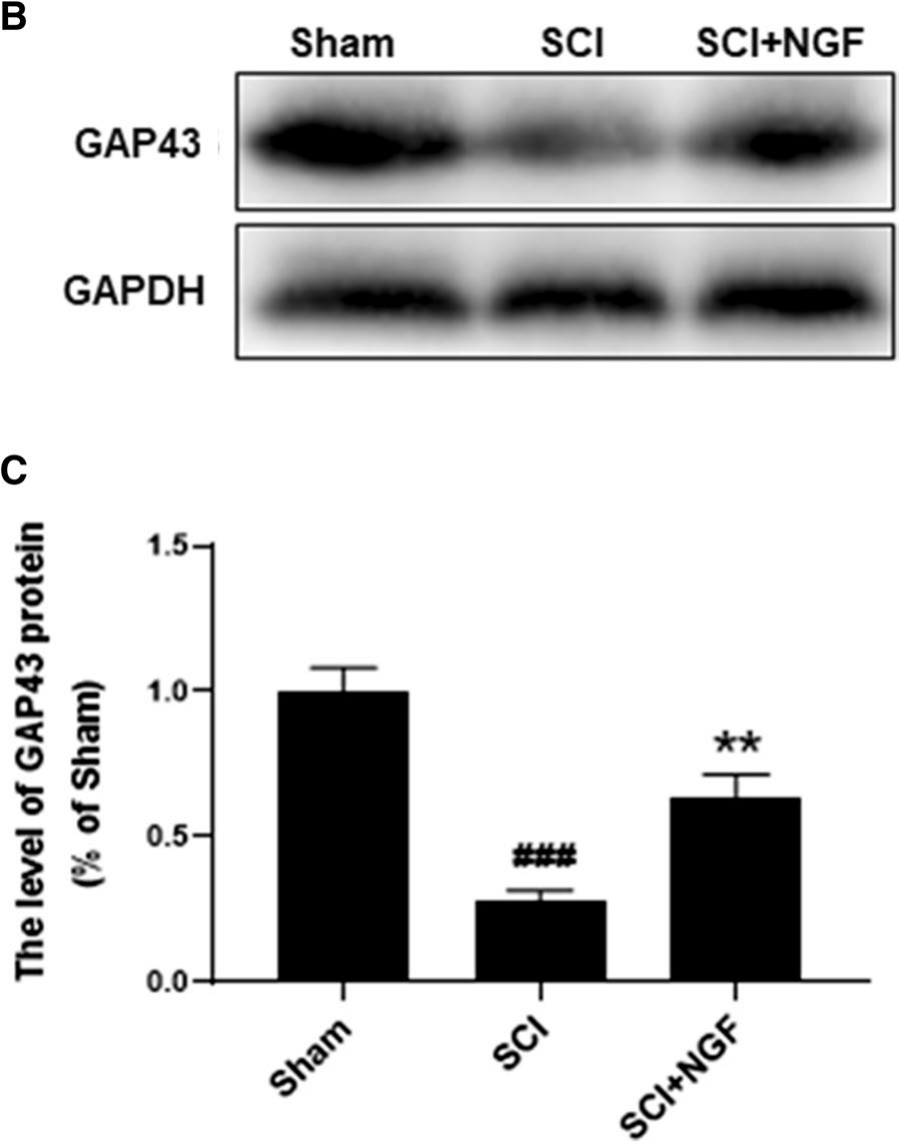
**B**. The protein expressions of GAP43 in sham, SCI rats and SCI rats treated with NGF groups. GAPDH was used as the loading control and for band density normalization. **C**. The optical density analysis of GAP43 protein. ** P < 0.01 versus the SCI group, and ### represents P < 0.005 versus the sham group. Data are the mean values ± SEM, n = 6

The effect of the nerve growth factor (NGF) group in promoting damage repair has not changed and the changes do not affect the scientific significance of current article.
